# Influence of Oral Dipping Tombak Smokeless Tobacco on Coagulation Profile and Platelet Counts

**DOI:** 10.3390/hematolrep14020019

**Published:** 2022-04-08

**Authors:** Ahmed M. E. Elkhalifa, Nada Y. Ali, Abdelhakam G. Tamomh, Mohammed I. Tabash, Esraa T. A. Mustafa, Zenieb A. K. Mohammed, Nedal A. S. Ahamed

**Affiliations:** 1 College of Health Sciences, The Saudi Electronic University, Riyadh 13323, Saudi Arabia; ahmedelnour2003@yahoo.com; 2 Faculty of Medical Laboratory Sciences, University of El Imam El Mahdi, Kosti 27711, Sudan; drnadoiayadeen80@gmail.com (N.Y.A.); sabahrahmttalla@gmail.com (E.T.A.M.); liuh9559@gmail.com (Z.A.K.M.); saharelamin60@gmail.com (N.A.S.A.); 3 Faculty of Applied Medical Sciences, Al-Azhar University, Gaza 1277, Palestine; mohtabash@gmail.com

**Keywords:** Tombak, prothrombin time, activated partial thromboplastin time, platelet counts, international normalized ratio, Sudan

## Abstract

The goal of this paper is to investigate the influence of oral dipping of Tombak Smokeless Tobacco (SLT) on prothrombin time (PT), activated partial thromboplastin time (APTT), international normalized ratio(INR) values, and platelet counts (PLTs), in Sudanese Tombak users. An analytical cross-sectional study was conducted at Kosti health insurance hospital, Sudan, in 2019. According to the inclusion and exclusion criteria, 100 adult users of oral Tombak for three or more years were chosen randomly as a study group. Another 100 matched healthy individuals who never used Tombak were randomly selected as a comparative group. Venous blood specimens were collected in ethylene diamine tetra-acetic acid (EDTA) containers for the PLT counts using the automated haematology analyser (Sysmex, Tokyo, Japan XK-21SYSMEX) and in trisodium citrate anti-coagulant containers for coagulation tests using a co-agulometer machine analyser. Our findings show a significant decrease in PLT count mean values in the Tombak users group (212.1 × 10^3^/mm^3^ ± 74.3 × 10^3^/mm^3^) compared with the non-taking Tombak group mean values (243.2 × 10^3^/mm^3^ ± 83.0 × 10^3^/mm^3^), (*p* < 0.006). Both PT and APTT were significantly prolonged in Tombak users (16.03 ± 1.22 s vs. 14.44 ± 0.557 s), *p* < 0.001 for PT, and (41.62 ± 7.28 s vs. 34.99 ± 4.02 s), (*p* < 0.001) for APTT. INR mean values were significantly longer in Tombak users (1.11 ± 0.096) vs. (1.07 ± 0.66; *p* < 0.001). Multiple linear regression analysis findings show a significant impact of the four investigated variables, including duration of taking Tombak, age, and frequency of taking Tombak per day (*p* < 0.001). In conclusion, using Tombak a Smokeless Tobacco (SLT) for a long period significantly affect Platelet counts and coagulation profile.

## 1. Introduction

The World Health Organization (WHO) reported 28.6% of the world’s population consumes tobacco, 10.7% of them in smoke form, and the other 21.4% usesmokeless tobacco (SLT), including snuffing, chewing, and dipping—either applied orally or inhaled through the nose [1,2,3]. Globally, more than 300 million people in more than 70 countries use SLT [4]. The tobacco epidemic is considered a major public health problem represented as one of the main disability and death causes worldwide; hence, the WHO estimated 8.3 million deaths related to tobacco use in 2030 and expected more than one billion deaths regarding tobacco use during the 21st century [2,5]. Tombak is a Sudanese locally made SLT dipping for oral users;it has been widely spread around the country for centuries among young adults and adolescents [6,7,8,9]. The prevalence range of Tombak use, according to previous surveys, is 34% among Sudanese adults males and about 25% among late adolescents, with a higher prevalence in rural areas [6,7,8,9]. All tobacco products, including SLT, contain nicotine, which is considered highly addictive. Nicotine enters the brain after being absorbed through the user’s mouth and continues to be absorbed into the bloodstream, and stays longer in the blood among SLT users than smokers [1]. Tombak is a moist substance with a strong, highly addictivearoma, pH range 8–11, moisture content 6–60%, and nicotine content from 8 to 102 mg/g wt [10]. However, the analysis of the chemical components of Tombak indicates 100-fold higher concentrations than tobacco-specific N-nitrosamines (TSNAs) of the commercial SLT brands from Sweden and the US [10]. The chemical substances found in Tombak have been associated with health hazards, including oral diseases and are reported to be responsible for oral cancer mucosal lesions, periodontal diseases, potentially malignant oral lesions, and may eventually lead to tooth loss [11,12,13]. Studies conducted among Sudanese who used Tombak confirmed a significant association between Tombak and oral cancer, with 3.2% incidence rates of oral cancer [14,15,16,17]. Furthermore, oral snuff use in Western Europe and North America is associated with cancer risk of the pharynx, oesophagus, pancreas, urinary bladder, kidney, leukoplakia preneoplastic changes oral, and nasal cavity cancer [14]. Various pharmacological actions of nicotine additives and their wide use in many countries and regions stated that the chronic consumption of SLT may influence the status of haematological profiles [1,18]. The effects of tobacco on the coagulation profile showed inhibition of production clotting factors, resulting in prolonged bleeding, which is attributable to coagulation factor inhibitors [19,20]. However, previous reports described the long-term harmful effects of SLT nicotine on various body parameters, but few studies examine the effect of consumption of SLT and little is known about the effect of Tombak on haematological parameters and coagulation profiles. Estimates of the prevalence and of Tombak use are scarce, so research on Tombak is very important regarding the impact and the magnitude of the problem. Therefore, our study aimed to investigate the influence of oral dipping Tombak SLT on coagulation profile and platelet counts in Sudanese attending Kosti health insurance hospitals who used Tombak SLT for three or more years continuously and compare the findings with the non-Tombak SLT results.

## 2. Materials and Methods

### 2.1. Study Design and Data Collection

An analytical cross-sectional study design was used and conducted at Kosti health insurance hospital, Sudan, in 2019. Two hundred males aged 20–65 years were randomly selected (100 oral Tombak users for three or more continuousyears and another 100 matched healthy individuals who never usedTombak).

Venous blood specimens were collected in the morning in ethylene diamine tetra-acetic acid (EDTA) containers for the PLT counts using the automated haematology analyser (Sysmex, Tokyo, Japan XK-21SYSMEX) and in tri-sodium citrate anti-coagulant containers for coagulation tests using a co-agulometer machine analyser.

### 2.2. Inclusion and Exclusion Criteria

Volunteer adult males who used Tombakfor three years or more and repeated taking Tombak three times or more daily were recruited in this study. Any disease in participants significantly affectedthe coagulation profile, liver diseases, hypertension, cardiovascular disease, a history of alcoholism, current drug effects on coagulation and platelet counts, and long-term medication (Heparin, Warfarin, and aspirin) or history of nonsteroidal anti-inflammatory drugs, and those who refused to participate were excluded. Similar mentioned excluded criteria applied to the control group.

### 2.3. Ethical Consideration

The study was approved by the Institutional Ethical Committee of the Faculty of Medical Laboratory Sciences, University of El Imam El Mahdi (Approval code No. EA.FMLS.UEE.2018-16. Approval date: 15 September 2018).

### 2.4. Statistical Analysis

The laboratory results and the demographic data were revised, coded, and proceed to SPSS Software (Statistical Package for the Social Sciences) version-25 (IBM Corp., Armonk, NY, USA). Frequency, percentage mean, and standard deviation were presented. A comparison between the two groups of participants applying *t*-test, one-way ANOVA, and multilinear regression methods. The two-tailed tests with an alpha error of ≤0.05 were considered statistically significant for a *p*-value ≤ 0.05.

## 3. Results

### 3.1. Demographic Characteristics

The study results indicated that the study population was entirely male. Oral Tombak SLT use in Sudan is a male-dominant habit, and females normally did not use Tombakor smoke cigarettes due to socio-cultural issues [7,21]. The age for the two groups varied from 20 to 65 years old, with a mean age of 39.46 ± 12.72 years old for the Tombak users group and 39.53 ± 12.21 years old for the non-Tombak users, and there was no statistically significant difference regarding age (*p*-value 0.968). In most of the Tombak users (66%), their duration of use was 3–5 years, (14%) for 6–7 years of use, and 3% used oral Tombak for more than ten years. Forty-eight per cent of the Tombak users repeated taking Tombak 3–5 times per day, 33% repeated taking Tombak 6–10 times per day, while the remaining 19% repeated taking Tombak more than ten times daily.

### 3.2. Effects of Taking Oral Tombak on Platelet Counts

**Figure 1:**
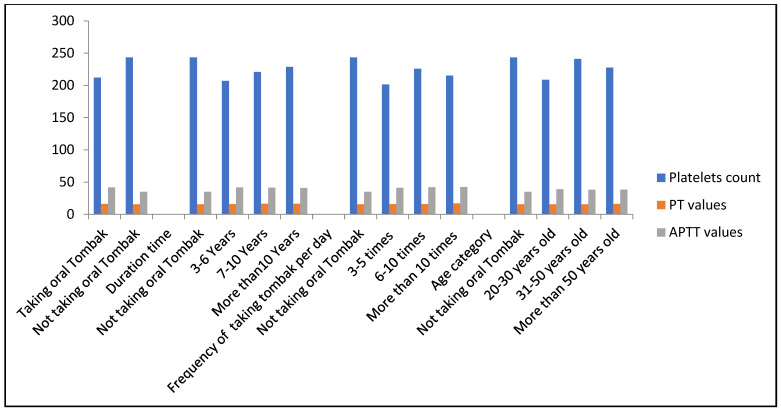
The effects of taking oral Tombak on PT, APTT, and PLT values among the study population.

As shown in Table 1 and Figure 1, therewas a significant decrease in the platelet count mean values among Tombak users (212.1 × 10^3^/mm^3^ ± 74.3 × 10^3^/mm^3^) compared with the non-taking Tombak group mean values (243.2 × 10^3^/mm^3^ ± 83.0 × 10^3^/mm^3^) (*p*-value = 0.0006). Furthermore, the results confirmed that the greaterduration time and the higher frequency of Tombakuse per day showed a decrease in plateletcount compared to the non-taking Tombak group with significant statistical differences (*p*-value = 0.037, 0.023, respectively). Tombak users who were more than 50 years old had lower platelet counts than the non-taking Tombak group with significant statistical differences (*p*-value = 0.0046).

### 3.3. Effects of Taking Oral Tombak on Prothrombin Time

The study results confirm a prolonged PT among the Tombak users group with a statistically significant differencecompared to the non-taking Tombak group (16.03 ± 1.22 s vs. 14.44 ± 0.557 s), *p*-value < 0.001 (Table 2 and Figure 1). The results show the same statistically significant difference regarding the duration of taking Tombak, age category, and frequency of taking Tombak per day, while the highest prolonged PT value was between Tombak users aged more than 50 years old.

### 3.4. Effects of Taking Oral Tombak on Activated Partial Thromboplastin Time

The results revealed a prolonged APTT mean value among the Tombakuser group (41.62 ± 7.28 s), showing a statistically significant difference when compared with the non-taking Tombak group (34.99 ± 4.02 s) (*p*-value < 0.001). However, the results show statistically significant differences regarding the duration of taking Tombak and frequency of Tombakuse per day; in contrast, there was no statistical difference concerning age category (Table 3 and Figure 1).

### 3.5. Effects of Taking Oral Tombak Based on International Normalised Ratio

The mean of INR in the used oral Tombak user group was longer than that of the non-taking Tombakgroup with a statistically significant difference (1.11 ± 0.096 vs. 1.07 ± 0.66; *p* < 0.001) (Table 4). The results showed the same statistically significant difference regarding the duration of taking Tombak, age category, and frequency of taking Tombak per day. The prolonged values were greater among participants aged > 50 years old (1.124 ± 0.106).

### 3.6. Effects of Taking Oral Tombak on PT, APTT, INR, and PLTs (Multiple Linear Regression)

As illustratedin Table 5, the multiple linear regression analysis findings show a significant impact of the mentioned four variables (PT, APTT, PLTs, and INR) regarding the duration of taking Tombak, age category, and frequency of taking Tombak per day. In the multiple linear regression, all of the compared items included (duration of taking Tombak, age category, frequency of taking Tombak per day), compared to other groups, showed a statistically significant decrease in the platelet counts, prolonged value of PT, and APTT values among the Tombak users group.

## 4. Discussion

The prevalence of Tombak use is popular among Sudanese male populations. It was found to be around 24% in urban areas and 35% in rural areas, with the highest rate among adolescents aged 18 years old and associated with cardiovascular disease and oral cancer [22]. There is a scarcity of data regarding the effect of Tombak dipping on the coagulation profile and platelet counts. Further efforts are needed to educate citizens with adverse health regarding dipping Tombak local habits. Therefore, our recent study findings are expected to provide baseline information regarding the influence of Tombak on coagulation profile and platelet counts. However, our results, compared with other studies conducted among heavier smokers and SLT, shared nicotine substance habits with Tombak users. Hence, Tobacco smoking and SLT are known to bea public problem worldwide among youths and have a significant role in coagulation malfunctions [21,23]. The objective of our current study was to determine platelet counts (PTLs), prothrombin time (PT), activated partial thromboplastin time (APTT), and INR values among Sudanese who partake in dipping Tombak orally three times or more daily for three years or more and compared their results with the non-users ofTombak. According to our findings, the platelet count mean values and multiple linear regression showed a statistically significant decrease among Tombak users compared tonon-Tombak users. Similar results in previous studies were reported with significantly lower platelet counts in chewing tobacco users, Shisha smoking, and heavier chronic smokers than non-smokers [21]. A study conducted by Oaikhena et al. [24] showed that the tobacco snuff could induce platelet count reduction among adult Wistar rats. In contrast, other previous studies concluded a significantly elevated platelet count in Tombak users, adult smokers, and tobacco chewers compared with control groups [24]. The PT mean values were found to be statistically significantly prolonged among the Tombak users group compared to the non-taking Tombak group. These results showed the same statistically significant difference regarding the duration of taking Tombak, age category, and frequency of taking Tombak per day. These findings are inconsistent with the previous studies completed by Metta et al. [25] and Al-Dahr [26], who reported no PT changes in smokers. However, studies by Ahmed Elkhalifa and Akpotuzor et al. [21,27] reported a significantly lower PT rate among smokers than the non-smoker participants. Furthermore, another study by Oaikhena et al. [24] showed a delay in PT among adult Wistar rats who used tobacco snuff. Concerning the APTT mean values, our findings show a prolonged APTT mean value among the taking Tombak group. These findings showed statistically significant differences regarding the duration of taking Tombak, frequency of taking Tombak per day, and non-statistical differences found concerning age category. Our results contrast a study by Akpotuzor et al. [27], who mentioned significantly lower APTT among chronic smokers. A study by Ahmed Elkhalifa [21] reported non-statistically significant differences between heavier smokers and non-smokers. Regarding the INR, mean values in the Tombak group werestatistically significantly longer than in the non-taking Tombak group. The results showed the same statistically significant difference regarding the duration of taking Tombak, age category, and frequency of taking Tombak per day. These current findings are inconsistent with the study by Nascetti et al. [28], who reported a decrease in INR value among cigarette smoking. A study conductedby Ahmed Elkhalifa [21] also stated a shorter INR value among the heavier smokers. Some of our results that were not in agreement with other studies might be due to the amount of nicotine components in Tombak SLT, the duration of taking Tombak, and the number of daily repeated uses ofTombak. The multiple linear regression analysis results showed a significant association of the four investigated variables in the current study (PLTs count, PT, APTT, and INR) with the duration of taking Tombak, age category, and frequency of taking Tombak per day.

## 5. Conclusions

Based on this study’s findings, Tombak SLT significantly decreases platelet counts, prolongs both PT, APTT, and has statistically significantly longer INR mean values. Moreover, the multiple linear regression analysis indicates a significant association between the coagulation profile measures and platelet counts concerning the duration of taking Tombak, age category, and frequency of taking Tombak per day. Further studies are recommended concerning the effects of Tombak SLT components on the coagulation profile and platelet counts to validate the current findings. Continuous educational and public awareness programs emphasising the adverse health effects of dipping oral Tombak is highly recommended.

## 6. Limitation of the Study

Our study focused on simple coagulation parameters (PT, APTT, INR) but also on platelet count. Further studies should be conducted to analyse the parameters of platelet function, red blood cell(RBCs) counts, and RBC indices.

## Figures and Tables

**Table 1:** The effects of taking oral Tombak on platelet counts among the study population.

Independent Variables	n	Mean	SD	t/F	*p*-Value
Taking oral Tombak	100	212.1 × 10^3^/mm^3^	74.3 × 10^3^/mm^3^	−2.789	0.0006 *
Not taking oral Tombak	100	243.2 × 10^3^/mm^3^	83.0 × 10^3^/mm^3^		
Duration time					
Not taking oral Tombak	100	243.2 × 10^3^/mm^3^	83.0 × 10^3^/mm^3^	−2.87	0.037 *
3–6 Years	66	206.9 × 10^3^/mm^3^	85.6 × 10^3^/mm^3^		
7–10 Years	28	220.8 × 10^3^/mm^3^	44.6 × 10^3^/mm^3^		
More than 10 Years	6	228.6 × 10^3^/mm^3^	46.8 × 10^3^/mm^3^		
Frequency of taking Tombak per day					
Not taking oral Tombak	100	243.2 × 10^3^/mm^3^	83.0 × 10^3^/mm^3^	−3.23	0.023 *
3–5 times	48	201.4 × 10^3^/mm^3^	97.1 × 10^3^/mm^3^		
6–10 times	33	225.9 × 10^3^/mm^3^	44.5 × 10^3^/mm^3^		
More than 10 times	19	215.1 × 10^3^/mm^3^	39.6 × 10^3^/mm^3^		
Age category					
Not taking oral Tombak	100	243.2 × 10^3^/mm^3^	83.0 × 10^3^/mm^3^		
20–30 years old	64	208.7 × 10^3^/mm^3^	115.3 × 10^3^/mm^3^	−3.12	0.046 *
31–50 years old	92	240.9 × 10^3^/mm^3^	60.4 × 10^3^/mm^3^		
More than 50 years old	44	227.5 × 10^3^/mm^3^	39.5 × 10^3^/mm^3^		

* Significance at the level ≤ 0.05; SD, standard deviation.

**Table 2:** The effects of taking oral Tombak on prothrombin time (PT) values among the study population.

Independent Variables	n	Mean	SD	t/F	*p*-Value
Taking oral Tombak	100	16.03 s	1.22 s	4.38	<0.001 *
Not taking oral Tombak	100	15.44 s	0.557 s		
Duration time					
Not taking oral Tombak	100	15.44 s	0.557 s	11.05	<0.001 *
3–6 Years	66	15.79 s	1.12 s		
3–7–10 Years	28	16.50 s	1.24 s		
More than 10 Years	6	16.41 s	1.57 s		
Frequency of taking Tombak per day					
Not taking oral Tombak	100	15.44 s	0.557 s	15.03	<0.001 *
3–5 times	48	15.83 s	1.06 s		
6–10 times	33	15.79 s	1.19 s		
More than 10 times	19	16.93 s	1.28 s		
Age category					
Not taking oral Tombak	100	15.44 s	0.557 s		
20–30 years old	64	15.60 s	0.911 s	5.02	0.007 *
31–50 years old	92	15.62 s	0.927 s		
More than 50 years old	44	16.14 s	1.14 s		

* Significance at the level ≤ 0.05. PT, prothrombin time; SD, standard deviation; s, seconds.

**Table 3:** The effects of taking oral Tombak on activated partial thromboplastin time (APTT)values among the study population.

Independent Variables	n	Mean	SD	t/F	*p*-Value
Taking oral Tombak	100	41.62 s	7.28 s	7.96	<0.001 *
Not taking oral Tombak	100	34.99 s	4.02 s		
Duration time					
Not taking oral Tombak	100	34.99 s	4.02 s	21.02	<0.001 *
3–6 Years	66	41.75 s	7.24 s		
7–10 Years	28	41.51 s	7.80 s		
More than 10 years	6	40.78 s	6.13 s		
Frequency of taking Tombak per day					
Not taking oral Tombak	100	34.99 s	4.02 s	21.18	<0.001 *
3–5 times	48	41.18 s	6.97 s		
6–10 times	33	41.96 s	7.55 s		
More than 10 times	19	42.15 s	7.88 s		
Age category					
Not taking oral Tombak	100	34.99 s	4.02 s	0.241	0.786
20–30 years old	64	38.77 s	6.64 s		
31–50 years old	92	38.01 s	6.78 s		
More than 50 years old	44	38.25 s	6.91 s		

* Significance at the level ≤ 0.05; APTT, activated partial thromboplastin time; SD, standard.

**Table 4:** The effects of taking oral Tombak based on the international normalised ratio (INR) value among the study population.

Independent Variables	n	Mean	SD	t/F	*p*-Value
Taking oral Tombak	100	1.11	0.096	3.59	<0.001 *
Not taking oral Tombak	100	1.07	0.066		
Duration time					
Not taking oral Tombak	100	1.07	0.066	8.68	<0.001 *
3–6 Years	66	1.09	0.077		
7–10 Years	28	1.15	0.120		
More than 10 Years	6	1.14	0.108		
Frequency of taking Tombak per day					
Not taking oral Tombak	100	1.07	0.066	12.5	<0.001 *
3–5 times	48	1.09	0.076		
6–10 times	33	1.09	0.077		
More than 10 times	19	1.19	0.130		
Age category					
Not taking oral Tombak	100	1.07	0.066	4.03	0.019 *
20–30 years old	64	1.0806	0.06700		
31–50 years old	92	1.0863	0.08291		
More than 50 years old	44	1.1243	0.10606		

* Significance at the level ≤ 0.05. INR, international normalisedratio.

**Table 5:** The effects of taking oral Tombak on PT, APTT, INR, and PLT values among the study population.

Variable	B	T/F	*p*-Value *	*p*-Value **
Platelet		2.77	0.043 *	
(Constant)	206.45	13.01	<0.001 *	
Age (category)	17.73	2.165	0.032 *	0.144
Duration time of taking Tombak	−3.908	−0.188	0.851	0.061
Frequency of taking Tombak	−11.37	−0.664	0.507	0.068
PT		11.66	<0.001 *	
(Constant)	15.25	82.47	<0.001 *	
Age (category)	0.090	0.941	0.348	0.010 **
Duration time of taking Tombak	0.064	0.265	0.791	<0.001 **
Frequency of taking Tombak	0.306	1.531	0.127	<0.001 **
APTT		19.45	<0.001 *	
(Constant)	39.04	32.61	<0.001 *	
Age (category)	−1.87	−3.034	0.003 *	0.641
Duration time of taking Tombak	−0.985	−0.628	0.530	<0.001 **
Frequency of taking Tombak	4.11	3.182	0.002 *	<0.001 **
INR		8.89	<0.001 *	
(Constant)	1.05	65.09	<0.001 *	
Age (category)	0.009	1.03	0.305	0.013 **
Duration time of taking Tombak	0.004	0.200	0.842	<0.001 **
Frequency of taking Tombak	0.023	1.33	0.185	<0.001 **

* *p*-value < 0.05, CI = 95% adjusted. ** *p*-value < 0.05, CI = 95% not adjusted.

## Data Availability

The data used to support the findings of this study are available from the corresponding author upon request.
